# Supplementary material for: GOLPH3-mTOR Crosstalk and Glycosylation: A Molecular Driver of Cancer Progression
Source: Cells. 2025 Mar 14;14(6):439. doi: 10.3390/cells14060439 (PMC11941073; doi:10.3390/cells14060439)
Supplement: Supplementary file 1 [file cells-14-00439-s001.zip › cells-3470496-supplementary.pdf]

Table S1. List of protein interactors of GOLPH3 described in the text.

PROTEIN	ORGANISM	FUNCTION	REF
GOLGI MAINTENANCE			
Myosin 18A	Human	• Linking Golgi to F-actin<br>• Generating a tensile force required for Golgi structure and trafficking	[4]
RAB1B	Human	PITPNC-RAB1B-GOLPH3 network leading to Golgi extension and enhanced vesicular release	[26]
GLYCOSYLATION			
Mannosyltransferases (Kre2, Mnn2, Mnn5, Och1)	Yeast	α-1,2-mannosyltransferase activity	[32]
Core 2 N-acetylglucosaminyltransferase 1 (C2GnT1)	Human	Synthesis of core 2-associated sialyl Lewis x	[34]
Protein O-Linked Mannose β-1,2-N-Acetlyglucosaminyltransferase 1 (POMGnT1)	Human	O-mannosylation of α-dystroglycan.	[35]
Exostosins EXT1 and EXT2	Fly	Synthesis of heparan sulfate proteoglycans	[38]
Exostosin EXTL3	Fly	Synthesis of heparan sulfate proteoglycans	[41]
Subunit of the oligosaccharyl transferase complex OstΔ	Fly	• N-glycosylation<br>• Initial transfer of Glc3Man9GlcNAc2 from dolichol-pyrophosphate to Asn-X-Ser/Thr consensus motif in the nascent polypeptides	[41]
α-1,2-mannosyltransferase (ALG11)	Fly	• N-glycosylation<br>• Addition of the first alpha-1,2-linked mannose residues	[41]
Phosphomannomutase type 2 (PMM2)	Fly	• N-glycosylation<br>• Converting mannose-6-phosphate to mannose-1-phosphate	[41]
Pgant5 and Pgant7	Fly	• Mucin-type O-glycosylation<br>• N-acetylgalactosaminyltransferase activity	[41]
Lactosylceramide synthase	Human	Converting glucosylceramide to lactosylceramide	[42]
β-Galactoside α-2,3-Sialyltransferase 2 (GD1a synthase)	Human	• Glycolipid glycosylation<br>• Ganglioside glycosyltransferase	[43]
β-1,3-galactosyltransferase 4 (GM1 synthase)	Human	• Glycolipid glycosylation<br>• Ganglioside glycosyltransferase	[43]
α-2,6-sialyltransferase-I (ST6GAL1)	Human	• N-glycosylation<br>• Terminal addition of sialic acid to glycans	[56]
mTOR			

VPS35	Human	• Retromer complex component<br>• Endosome-to-Golgi trafficking	[7]
Rheb	Fly	mTORC1 pathway	[80]
Tctp	Fly	mTORC1 pathway	[80]
14-3-3ζ	Fly	mTORC1 pathway	[80]
Lst8	Fly	mTORC1/mTORC2 pathway	[80]
CYTOKINESIS			
Rab1	Fly	• Rab GTPase<br>• ER to Golgi and intra-Golgi trafficking	[88]
Zipper/NMII heavy chain	Fly	• Structural component of the contractile ring	[90]
Pavarotti/MKLP1	Fly	• Centralspindlin component<br>• Positioning of the contractile ring	[90]
